# An Italian National Survey on Ovarian Cancer Treatment at first diagnosis. There's None so Deaf as those who will not Hear

**DOI:** 10.7150/jca.57894

**Published:** 2021-05-27

**Authors:** Vincenzo Dario Mandato, Federica Torricelli, Stefano Uccella, Debora Pirillo, Gino Ciarlini, Gabriele Ruffo, Gianluca Annunziata, Gloria Manzotti, Sandro Pignata, Lorenzo Aguzzoli

**Affiliations:** 1 Unit of Obstetrics and Gynecology, Azienda USL-IRCCS di Reggio Emilia, Reggio Emilia, Italy.; 2 Laboratory of Translational Research, Azienda USL-IRCCS di Reggio Emilia, Reggio Emilia, Italy.; 3 Department of Obstetrics and Gynecology, AOUI Verona, University of Verona, Verona VR, Italy.; 4 Unit of Surgical Gynecol Oncology, Azienda USL-IRCCS di Reggio Emilia, Reggio Emilia, Italy.; 5 Istituto Nazionale Tumori Fondazione Giovanni Pascale, Istituto di Ricovero e Cura a Carattere Scientifico, Napoli, Italy.

**Keywords:** epithelial ovarian cancer, centralization, survey, gynecologic oncologists, cytoreduction

## Abstract

**Objective:** Epithelial ovarian cancer (EOC) is the most lethal gynecological malignancy, crucial prognostic factors are no gross residual disease and centralization of cases. To evaluate the centralization of EOC patients, we report the results of a survey that shows the daily management of EOC patients in Italy.

**Methods:** A 49-items electronic unblinded survey assessing demographics, practice characteristics, current opinions and approach to managing advanced EOC at first diagnosis was sent both to general gynecologists (GG) and gynecologic oncologists (GO). Differences in frequency distribution of answers between gynecologists with different expertise were evaluated using Fisher exact test. Multivariable analyses were performed applying generalized linear models.

**Results:** 84/192 (44%) GG and 108/192 (56%) GO from all Italian regions answered to our survey. GOs declared to perform fertility sparing surgery in early EOC more frequently than GG (p=0.002).

GOs can perform a frozen section and have both a gynecopathologist and a dedicated general surgeon. 89% of GOs consider as “optimal debulking” no gross residual disease and 81% achieve this at upfront cytoreduction in more than 40% of patients. Use of neoadjuvant chemotherapy decreases in higher volume centers (p<0.001) while it is lower in the group of GOs than in the GGs group (p<0.001).

**Conclusions:** EOC patients are still treated by GGs. GOs perform more upfront surgery and achieve optimal debulking in a greater percentage of patients than GGs. In Italy an adequate centralization of cases has not yet been achieved, and this may have detrimental effects on the quality of treatment.

## Introduction

Epithelial ovarian cancer (EOC) is the most lethal gynecological malignancy, with 238,700 new cancer cases and 151,900 cancer deaths worldwide recorded in the 2012 1. This malignancy represents the eighth cause of death from cancer in women worldwide 1. In the year 2016, the Italian Association of Cancer Registries reported 5,200 new cancer cases and 3,302 cancer deaths 2. Despite well-recognized advances in treatment 3-12, overall mortality rate has not substantially improved 13. Five-year overall survival (OS) is generally low, being around 45% 14 because EOC is usually diagnosed at an advanced stage and no specific symptoms or screening tools are available for early diagnosis 7,14,15. When EOC is limited to the ovary (stages IA and IB), 5-year OS rises to 92%. However, only 15% of all EOC are found at these early stages 14, therefore the diagnosis of an EOC is often associated with a heavy impact on women's life 16,17.

Although new prognostic factors are continuously researched 7,18-22 and others such as age, obesity, performance status, histology, stage, and grade are well established 18, the most important aspects associated with improved survival are the absence of macroscopic residual disease at the end of primary surgery and centralization of cases 18,23. Several reports and meta-analysis provide evidence to suggest that EOC patients who receive treatment in high volume and specialized centers have a significantly improved survival compared to those managed elsewhere 13, 23-30. High volume centers usually guarantee high-quality surgery, maximal cytoreduction and very high rates of no residual disease 23, 26-28. Usually EOC patients are consistently treated according to established guidelines at high volume centers 31 and when better surgical results are achieved EOC treatment becomes cost-effective 32. Despite this compelling evidence, many EOC patients are not referred to high-volume, specialized centers 33. In 2013, an Italian regional audit concerningly reported that most of the hospitals (84%) treating EOC patients were low volume centers (≤ 10 operated patients/year) and managed 22.3% of EOC patients, while only 45.6% of EOC patients were treated in high volume EOC centers (≥ 21 patients/year) 23. These differences translated into a significantly better survival among patients managed at high-volume centers 23. Similar findings have been described by a recent report of The Italian National Agency for Regional Healthcare Services (AGENAS) which showed that 316/415 (76%) hospitals treat less than 10 EOC patients per year, while only 50/415 (3.3%) hospitals manage more than 20 EOC patients for year 34.

With the purpose of understanding whether there has been an improvement in the centralization of the cases, we report the results from a survey aimed at drawing a true picture of the daily management of EOC at primary diagnosis in Italy.

## Materials and methods

A 49-items electronic unblinded survey (Table 1) assessing demographics, practice characteristics, and current opinions and approach to managing advanced EOC at first diagnosis was sent to the Multicenter Italian Trials in Ovarian Cancer and Gynecologic Malignancies (MITO) Group members, Menopausa-Italia Group members and was published on the website of the Italian Association of the Ostetricians and Gynecologists (AOGOI). The results were collected by using Google Forms available at this link: https://docs.google.com/forms/d/1zeXslexB7ODS2x9MQs-hCHC8H8X_nE2HFCPvYhaq-wY/edit. Gynecologists received an email three times with a link to the survey and the free access to the survey link was available on AOGOI web site for three months (https://www.aogoi.it/notiziario/indagine-conoscitiva-sul-trattamento-delle-pazienti-con-prima-diagnosi-di-carcinoma-ovarico/). Participants had to answer to all questions and had the possibility of receiving the result of the questionnaire. The survey was addressed both to general gynecologists and to gynecologists with specific interest in gynecologic oncology. In Italy no subspecialty or formal fellowship in Gynecologic Oncology exists; therefore, we defined the General Gynecologists (GGs) as those gynecologists who are involved in various aspects of Obstetrics and Gynecology during their clinical practice and who occasionally treat malignancies. On the other hand, gynecologists with specific interests in gynecologic oncology (GOs) were defined as those gynecologists who spend the majority of their clinical practice in the treatment of gynecologic malignancies. The first survey request was sent out with an e-mail invitation and link to the survey in April 2019, with a second invitation sent to non-responders three weeks later, and a third and final invitation sent four weeks later. To ensure the validity and reliability of the survey, we followed the recommendations by Tong et all 35. Demographics of the surveyed cohort were analyzed using R software version 2.15.1. Differences in frequency distribution of survey answers between medical doctors with different expertise were evaluated using Fisher exact test. Multivariable analyses were performed applying generalized linear models. Statistically significant differences were expressed by a *P* value lower than 0.05.

## Results

From 250 surveys sent out 226 replies were included in the final analysis (return rate 90.4%): 34/226 residents (15%) and 192/226 (85%) specialists (Supplementary Table 1) answered. Answers came from different Italian regions and were sorted in three big area such as Northern, Central, and Southern Italy. This study was focused on specialists' point of view and all the following data were based on the answers of these 192 physicians (Table 1).

Among specialists, 84/192 (44%) of the respondents are GGs and 108/192 (56%) are GOs. Half of the GO (54/108) are over 50 years old and 70% of them (76/108) have been working for more than fifteen years. 74% of GOs treat EOC surgically as first operator (FS), while 64% (54/84) of GGs operate as assistant (AS) (Table 2). Gynecologists who work in a center treating 10 EOC patients/year or less are occasionally dedicated to gynecologic oncology in 85% of cases (44/52); on the contrary, in centers treating more than 30 EOC patients/year, gynecologists are dedicated to gynecological oncology as their main activity in 86% of cases (62/72) (Table 2).

**Figure 1:**
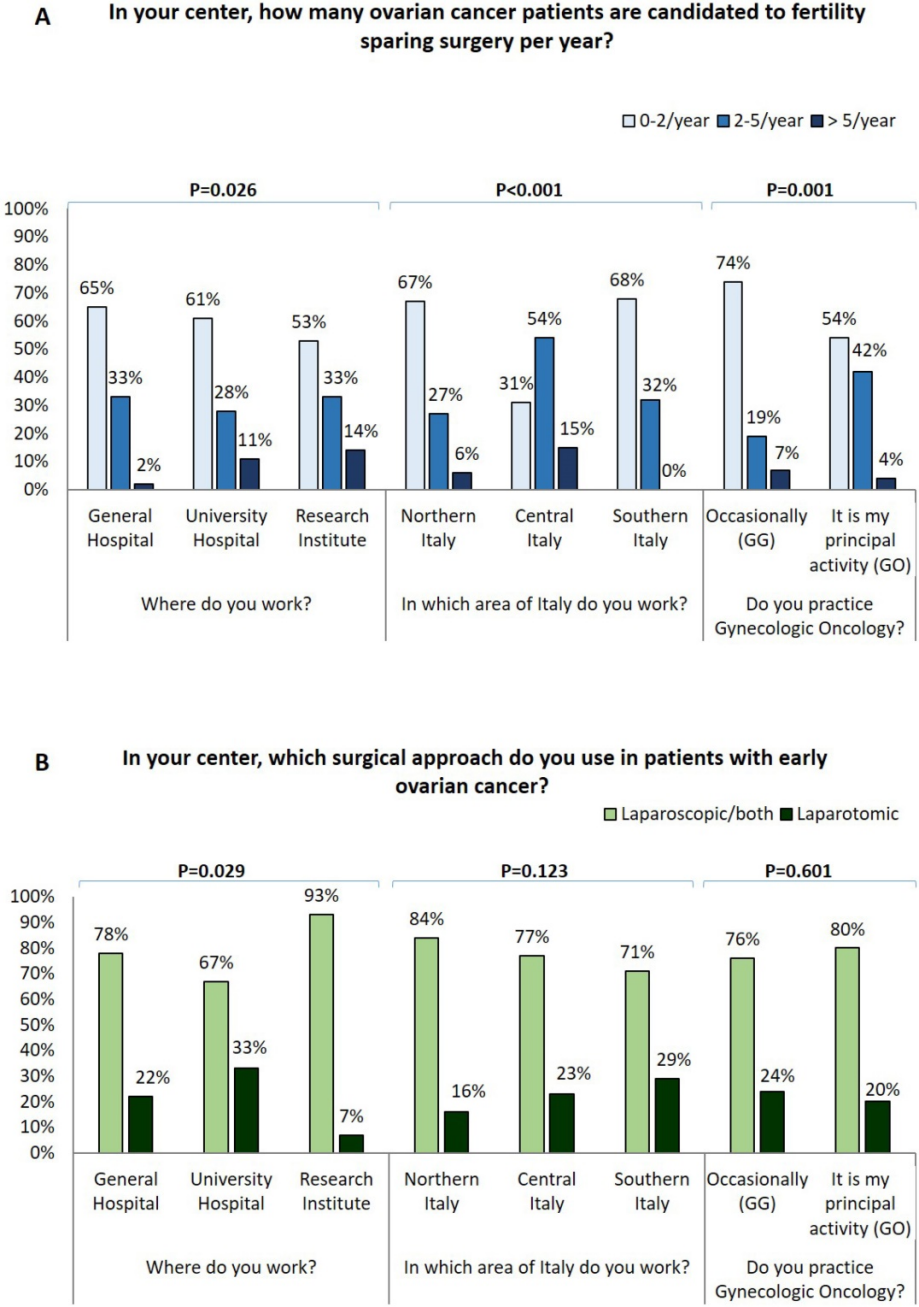
A) Influence of surgical centre, geographical area, oncological practice on number of ovarian cancer patients candidate to fertility sparing surgery for year; B) Influence of surgical centre, geographical area, oncological practice on surgical approach used in early stage ovarian cancer patients.

GOs declared to perform fertility sparing surgery in early EOC more frequently than GG (p=0.002) (Figure 1A). In particular, a higher number of fertility-sparing approaches per year was reported by specialists from central Italy and both laparoscopy and fertility sparing were particularly employed in research institutes (Figure 1A-1B).

All GOs who participated to our survey work in centers where there is the possibility to perform an intraoperative frozen section and in more than 70% of cases a pathologist with particular expertise in gynecological oncological pathology (gynecolopathologist) and a general surgeon dedicated to EOC treatment are available (80% and 74% respectively); conversely GGs declared the availability of dedicated gynecopathologist and a general surgeon only in 48% and 52% of cases respectively.

**Figure 2:**
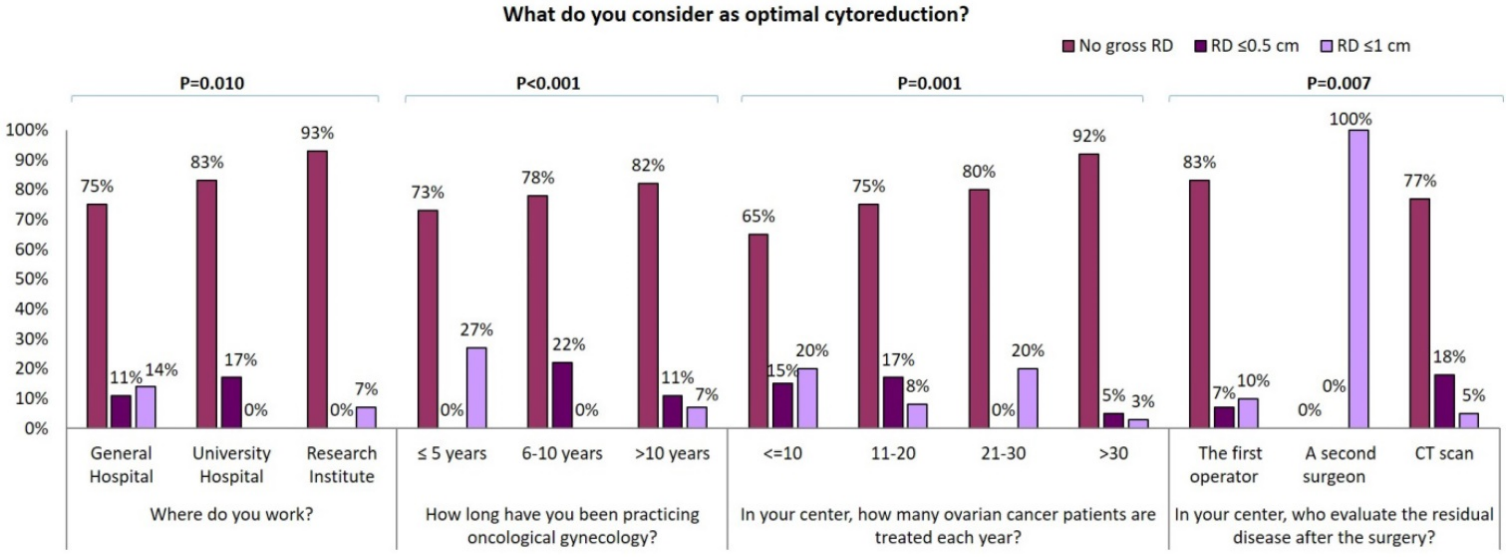
Influence of surgical centre, years of experience in oncological gynecology, number of ovarian cancer patients treated for year and modality of residual tumor evaluation on optimal cytoreduction definition.

The vast majority of GOs (96/108, 89%) consider as “optimal debulking” no gross residual disease (RD) and 88/108 (81%) achieve this at upfront cytoreduction in more than 40% of patients. Conversely, 67% (56/84) of GGs occasionally dedicated to gynecologic oncology, consider as “optimal debulking” no gross RD whilst 19% (16/84) of them consider as “optimal debulking” RD ≤ 1 cm. Besides, less than half of GGs declared to achieve complete cytoreduction in more than 40% of patients. Particularly, “optimal debulking” is considered as no gross RD by 93% of responders working at Research centers, 83% of responders working at University centers and only 75% of responders working at general hospitals. Interestingly, the percentage of this answer raises with the increase of the years of experience in gynecological oncology and the number of EOC patients treated each year in the center (Figure 2A) Moreover, the RD is evaluated by the first operator in 75% of cases; 24% of the responding specialists utilize a postoperative CT scan to evaluate the completeness of cytoreduction; 1% of responders declared that completeness of cytoreduction was evaluated by a second surgeon (Table 1). Those declaring that gross RD is defined by the first operator consider no gross RD as optimal debulking in a larger percentage of cases (Figure 2).

GGs who answered to our survey performed less upper abdominal surgery than GOs (Table 2). 69% of GGs who answered to the survey never perform a diaphragmatic resection while 52% of GOs perform a diaphragmatic resection in 1-20% of EOC and 20% declare to perform it in a higher percentage of patients. Similarly, 52% of GGs never perform a diaphragmatic peritonectomy in comparison with only the 11% of GOs.

**Figure 3:**
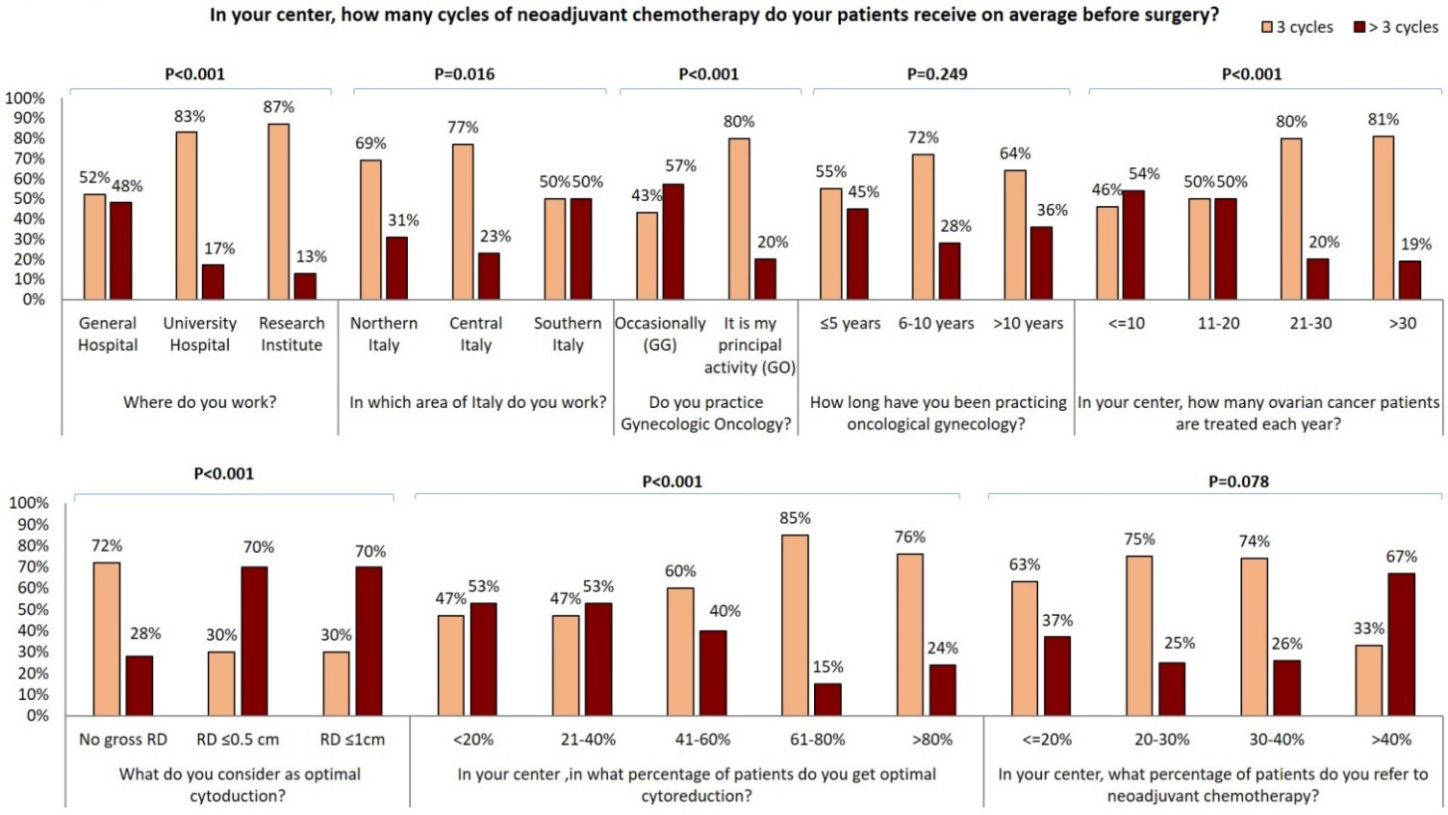
Influence of surgical centre, geographical area, oncological practice, years of experience in oncological gynecology, number of ovarian cancer patients treated for year, optimal residual tumor definition, percentage of optimally cytoreducted patients and percentage of patients submitted to neoadjuvant chemotherapy on number of neoadjuvant chemotherapy cycles.

In our survey 72% of GOs and only 24% of GGs perform bowel resection in more than 5% of EOC cases. Moreover, 60% of GGs never perform hepatic surgery and 38% perform it in less than 10% of EOC. Conversely, 80% of GOs declare to carry out hepatic surgery in 1-10% of patients and 7% perform it in more than 10% of patients. Similar data were collected about other surgical practices: 76% of GGs versus 46% of GOs never perform a distal resection of the pancreas while cholecystectomy is never carried out by 45% of GGs versus 17% of GOs. Systematic pelvic and para-aortic lymphadenectomy are never carried out by 37% and 44% of GOs respectively, versus 14% and 17% of GG. In fact, 43% of GOs and only 14% of GGs perform the resection only of gross lymph nodes in 75-100% of patients. 72% of the responders administer neoadjuvant chemotherapy (CHT) to more than 20% of patients: 83% of responders administer carboplatin and paclitaxel while carboplatin-only therapy is delivered only by 10 responders in Northern Italy (Table 1). 64% (122/192) of responders administer three cycles of neoadjuvant CHT while >3 cycles are administered by majority larger percentage of specialists from southern Italy (Figure 3). Of note, gynecologists who achieve optimal cytoreduction in at least 40% of patients declare to adopt an approach of neoadjuvant CHT in a lower percentage of cases and to administer 3 cycle of therapy in the majority of cases. Conversely, 70% of those gynecologists who consider optimal RD ≤1 cm declare to administer more than 3 cycles of chemotherapy (Figure 3). Moreover, specialists who treat more than 40% of EOC patients with neoadjuvant chemotherapy administer more than 3 cycles of neoadjuvant CHT in 67% of cases. 48% of physicians working in general hospital administer more than 3 cycles of CHT in comparison with 17% and 13% of those working in university hospitals and research institutes respectively (p<0.001). Besides, the percentage of gynecologists who administer more than 3 cycles of CHT in the neoadjuvant setting decrease in centers where a larger number of EOC are registered each year (p<0.001) and is lower in the group of GOs than in the GGs group (20% vs 57%, p<0.001). Moreover, the percentage of gynecologists who administer more than 3 cycles of neoadjuvant CHT shows a decreasing trend among physicians with a longer experience in gynecologic oncology and correlation between experience in gynecologic oncology and number of neoadjuvant CHT cycles became statistically significant (p=0.017) comparing specialist who administer ≤ 4 cycles with specialist administering > 4 cycles. 110/192 (57.3%) specialists declared to operate as first surgeon (FS) and 82/192 (42.7%) as assistant (AS) (Supplementary Table 2). ASs are significantly younger and less experienced that FSs, 34% of ASs versus 73% of FSs declared to practice gynecologic oncology as principal activity. 37% of ASs versus 20 % of FSs (P=0.045) work in center where no more than 10 cases of EOC are treated each year. ASs more frequently work in centers were only 0-2 patients per year are candidate to fertility sparing (73% of ASs vs 55% of FSs, P=0.002) and were laparoscopy is never adopted in patients candidate to laparoscopy (24% of ASs vs 7% of FSs, P=0.010). 51% of ASs versus 75% of FSs has a dedicated general surgeon in his team. 48% of ASs declared to obtain an optimal cytoreduction in less than 40% of patients while only 28% of FSs has the same opinion (P=0.002). ASs in comparison to FSs declared to work in center where less aggressive surgery is performed: they never perform diaphragmatic resection (61% vs 35%), diaphragmatic peritonectomy (42% vs 22%), liver resection (51% vs 20%), distal resection of the pancreas (71% vs 51%) and cholecystectomy (37% vs 24%) and where less than 5% of EOC patients receive bowel resection (71% vs 33%) and splenectomy (80% vs 55%). On the contrary, 37% of ASs versus 16% of FSs (P=0.018) and 27% versus 11% (P=0.016) declared to perform respectively systematic pelvic and lombo-aortic lymphadenectomy in more than 50% of patients. Similarly, 37% of ASs declared to only perform bulky lymph nodes removal in less than 25% of cases versus only 13% of FSs. Furthermore, only 12% of ASs versus 24% of FS (P=0.011) work in centers where more than 40% of patients receive neoadjuvant chemotherapy but 46% of ASs and only 27% of FSs declare that patients treated with chemotherapy receive more than 3 cycles of treatment.

## Discussion

The present survey was created to assess how EOC patients are treated in Italy in the everyday clinical practice and to understand which geographic, professional or clinical variables influence the physicians' therapeutic choices. Among the other findings, the present national analysis shows that in 2020 there is a wide variation of treatment policies for EOC patients between dedicated gynecologic oncologists and general gynecologists and that still a vast proportion of gynecologists in Italy treat ovarian cancer in low-volume hospitals.

We focused our data analysis on the perspective of the 192 responding specialists and we based the principal comparisons on the differences between GGs and GOs.

Initially, we observed that the percentage of physicians mainly dedicated to gynecologic oncology is higher in centers were a higher number of EOC are treated each year. This data could suggest some considerations: the first is that centers with small numbers of EOC patients do not usually treat gynecological cancers, while probably gynecologists who work in higher-volume centers are also likely to treat more patients with gynecological cancers other than the ovary.

Considering only specialist ASs, most are younger, less experienced, work in low volume center and are occasionally dedicated to gynecologic oncology so most of their answers resemble with those of GGs. Notably, we observed that GOs perform fertility-sparing surgery in a significantly larger percentage of early EOC than GGs. Of note, both fertility-sparing surgery and laparoscopic approach are used more commonly at research centers. This is in line with international guidelines which suggest the performance of laparoscopy and fertility sparing procedures only at referral centers 36. It is essential to note that in younger patients with early EOC, expert GOs can perform a less invasive approach but also a less aggressive surgery ensuring a complete staging and preserving fertility.

EOC treatment requires an interdisciplinary approach and multidisciplinary team that should include GOs, experienced and dedicated general surgeons, medical oncologists, radiation oncologists, radiologists, pathologists, psychologists, nutritionists and researchers 33,37. In our survey, the majority of GOs work in centers where there is always the possibility to perform an intraoperative frozen section and where a gynecopathologist and a general surgeon dedicated to EOC treatment are generally available; on the other hand, only a small proportion of GGs declare to have the possibility of such a multidisciplinary collaboration.

Several studies have shown that cytoreduction < 1 cm RD provides relevant survival benefits and that no gross RD at the end of initial surgery is associated with significantly longer overall survival, compared to suboptimal cytoreduction 38,39. In our survey, almost all GOs consider as “optimal debulking” no gross RD while only 67% of GGs is of the same opinion and declare to achieve complete cytoreduction at up front surgery. Interestingly, the percentage of specialists that consider no gross RD as “optimal debulking” is higher among those who work in research centers, have a longer expertise or manage a higher number of EOC per year. On the other hand, most specialists who consider as optimal a RD ≤1 cm work in general hospitals, regardless of age, experience and time spent for gynecological oncology.

It has been clearly demonstrated that the rate of optimal cytoreduction can be sensibly improved by inclusion of upper abdominal procedures 40. Consequently, cancer centers routinely offer splenectomy, diaphragm resection, celiac nodal resection, and/or multiple bowel resections, cholecystectomy, partial pancreatectomy 41-45. Notably, GGs who answered to our survey perform less upper abdominal surgery than GOs: about 70% of GGs versus 30% of GOs never perform diaphragmatic resection and similarly about half of GGs never perform a diaphragmatic peritonectomy in comparison with only a small percentage of GOs. Liver metastases account for 18% of parenchymal disease and have been described as the second most common cause of stage IV EOC in a large GOG study 41,47. Complete resection rates vary from 56 to 87.5% among studies reported in the literature 48. Complete liver metastases resection seems to depend on metastatization pattern; it has been proposed that metastases due to hematogenous spread should be submitted to neo-adjuvant chemotherapy whilst metastases due to transcoelomic seeding could be successful resected 48. Interestingly, in our survey, 60% of GGs and only 13% of GOs declared that they never perform hepatic surgery. Bowel resection is one of the most common procedures performed to achieve optimal RD and is estimated that it is required in approximately 50% of optimal cytoreductive operations 49. 76% of the responding GGs carry out bowel resection in < 5% of EOC while the vast majority of GOs perform it in a high percentage of cases. Moreover, the greatest number of procedures on the upper abdomen are reported by physicians working in centers of central and northern Italy. Lymphatic spread is a common finding and an important prognostic factor in both early and advanced EOC 50. Recently, the LION trial indicated that systematic lymphadenectomy does not offer a survival benefit in advanced EOC patients with no gross lymph node metastases, and that paraaortic and pelvic lymphadenectomy is still warranted in macroscopically suspicious nodes to achieve complete cytoreduction 50. Within our cohort of specialists, about 40% of GOs and 15% of GGs never perform systematic pelvic and para-aortic lymphadenectomy; similar percentages of responders perform only resection of gross lymph nodes in 75-100% of patients. Multivisceral resections should be performed only if a complete cytoreduction with absent RD can be achieved 37. In our survey, RD is judged in a large majority of cases by the first operator and only a small number of specialists evaluate it using postoperative CT scan. Diagnostic laparoscopy was suggested as a feasible approach to assess intraperitoneal diffusion of EOC and the likelihood of complete cytoreduction 51. In our cohort, no significant differences in the use of laparoscopic approach was observed between GOs and GGs. Despite upfront debulking surgery remains the best treatment of advanced EOC, since the publication of the EORTC 55971 trial 52 and CHORUS trial 53 many centers promote the use of neoadjuvant chemotherapy associating it to a reduction in surgical morbidity. A concern about a policy of systematic adoption of diagnostic laparoscopy as a triage for patients with advanced EOC is that it may be used by less experienced gynecologists; the consequence of this policy may be to send too many patients to neoadjuvant chemotherapy, in order to perform a potentially less demanding interval debulking surgery. Most of the gynecologists who answered our survey adopt neoadjuvant therapy in more than 20% of EOC cases and administer a combined carboplatin-paclitaxel chemotherapy, while only a small number of responders, from northern Italy, use carboplatin-only chemotherapy. Generally, approximately 40% of EOC patients present with malnutrition, bowel dysfunction, extensive upper abdominal or extraperitoneal disease, large-volume ascites, advanced age and associated comorbidities. Many of these EOC patients will receive neoadjuvant CHT with consideration of interval cytoreductive surgery 54. The majority of the responding specialists established the appropriate number of neoadjuvant CHT cycles as three, but this number is often higher for physicians who candidate more patients to neoadjuvant therapy. The percentage of physicians that administer more than 3 cycles of neoadjuvant CHT varies between the different clinical centers and between different areas of Italy as well as is higher among specialists who occasionally practice gynecological oncology or those who work in centers where a small number of EOC is treated. On the other hand, the percentage of gynecologists administering more than 3 neoadjuvant CHT cycles is lower between those who consider no gross RD as optimal debulking and who declare to obtain complete cytoreduction in a larger percentage of patients.

This survey highlights how the figure of the GO, particularly FSs, and of the other specialists dedicated to the treatment of ovarian cancer is fundamental to guarantee the best treatment for EOC patients. Compliance with the quality indicators such as percentage of up-front surgery, of optimal RD achieved, of patients submitted to neoadjuvant chemotherapy, number of cycles of neoadjuvant chemotherapy, use of minimally invasive surgery can only be obtained by managing high volumes of EOC patients and with continuous training. Although many studies and guidelines have highlighted the need to centralize the treatment of ovarian cancer in high volume centers, it seems that this recommendation is not yet respected in a vast proportion of cases.

Even the blindly obvious is never too obvious for everyone. Knowing the real world is essential to start promoting changes in the treatment of EOC patients. Despite the fact that since 2004 the Italian national guidelines program has supported centralization for EOC patients 55, several authors over time have highlighted a lack of centralization 56 which still seems to persist (Table 1) and that likely reflects into a suboptimal treatment of affected patients. The national health system, the scientific societies and first of all the individual specialists (gynecologists, oncologists, general surgeons, general practitioners) should guarantee all patients the most appropriate treatments by directing them to the centers with greater competence. In the absence of a structured cancer network, the individual specialist should voluntarily centralize the patient to the nearest competent center avoiding inadequate treatments and wasted time that could have a detrimental impact on survival.

## Supplementary Material

Supplementary tables.

## Figures and Tables

**Table 1:** Frequency distribution of survey questions and answers of specialists

	Total (N=192)
**1. Where do you work?**	
a. General Hospital	126 (66%)
b. University Hospital	36 (19%)
c. Research Institute	30 (16%)
d. Private Clinic	0 (0%)
**2. In which area of Italy do you work?**	
a. Norther Italy	98 (51%)
b. Central Italy	26 (14%)
c. Southern Italy	68 (35%)
**3. You are:**	
a. Resident	0 (0%)
b. Specialist	192 (100%)
**4. How old are you?**	
a. < 30 years	0 (0%)
b. 30-35 years	24 (12%)
c. 36-40 years	28 (15%)
d. 41-50 years	50 (26%)
e. > 50 years	90 (47%)
**5. How many years of practice do you have?**	
a. ≤ 5 years	28 (15%)
b. 6-10 years	18 (9%)
c. 11-15 years	26 (14%)
d. > 15 years	120 (62%)
**6. Do you practice Gynecologic Oncology?**	
a. Occasionally	84 (44%)
b. It is my principal activity	108 (56%)
**7. Do you perform EOC surgery as:**	
a. First surgeon	110 (57%)
b. Assistant surgeon	82 (43%)
**8. How long have you been practicing oncological gynecology?**	
a. ≤ 5 years	44 (23%)
b. 6-10 years	36 (19%)
c. > 10 years	112 (58%)
**9. In your center, how many ovarian cancer patients are treated each year?**	
a. ≤10	52 (27%)
b. 11-20	48 (25%)
c. 21-30	20 (10%)
d. >30	72 (38%)
**10. In your center, how many ovarian cancer patients at stage I-II are treated each year?**	
a. ≤5/year	66 (34%)
b. 6-10/ year	74 (39%)
c. 11-15/year	26 (14%)
d. 15/ year	26 (14%)
**11. In your center, which surgical approach do you use in patients with early ovarian cancer?**	
a. Laparoscopic/both	150 (78%)
b. Laparotomic	42 (22%)
**12. In your center, in what percentage of cases do you use the laparoscopic approach?**	
a. ≤10	56 (29%)
b. 11-30	54 (28%)
c. > 30%	82 (43%)
**14. In your center, how many ovarian cancer patients are candidated to fertility sparing surgery each year?**	
a. 0-2/year	120 (62%)
c. 2-5/year	62 (32%)
b. > 5/year	10 (5%)
**18. In your center, which surgical approach do you use in patients with ovarian tumors who are candidates for fertility sparing surgery?**	
a. Laparoscopic/both	170 (89%)
b. Laparotomic	22 (11%)
19. In your center, in what percentage of cases do you adopt the laparoscopic approach in patients candidated for fertility sparing surgery?*	
a. 0%	26 (14%)
b. < 25%	60 (33%)
c. 25-50%	22 (12%)
d. 50-100%	50 (27%)
e. 100%	26 (14%)
**21. In your center, do you have the opportunity to perform an extemporaneous intraoperative examination?**	
a. Yes	186 (97%)
b. No	6 (3%)
**22. In your center, do you have a dedicated pathologist available?**	
a. Yes	126 (66%)
b. No	66 (34%)
**23. Do you have a dedicated general surgeon on your team?**	
a. Yes	124 (65%)
b. No	68 (35%)
**24. What do you consider as optimal cytoduction?**	
a. No gross residual disease	152 (79%)
b. Residual disease ≤ 0.5 cm	20 (10%)
c. Residual disease ≤ 1 cm	20 (10%)
**25. In your center, who evaluate the residual disease after the surgery?**	
a. The first operator	138 (75%)
b. A second surgeon	2 (1%)
c. The patient undergoes a CT scan after the surgery	44 (24%)
**26. In your center, in what percentage of patients do you get optimal cytoreduction?**	
a. <20%	34 (18%)
b. 21-40%	34 (18%)
c. 41-60%	50 (26%)
d. 61-80%	40 (21%)
e. >80%	34 (18%)
**28. In your center, in cases where you suspect the impossibility of direct cytoreduction, do you always perform a diagnostic laparoscopy before laparotomy?**	
a. Yes	154 (80%)
b. No	38 (20%)
**29. In your center, in cases where you suspect the impossibility of direct cytoreduction, do you always perform a minilaparotomy before laparotomy?**	
a. Yes	28 (15%)
b. No	164 (85%)
**31. In your center, in what percentage of patients do you perform a diaphragmatic resection?**	
a. 0%	88 (46%)
b. 1%-20%	76 (40%)
c. 21-40%	12 (6%)
d. >40%	16 (8%)
32. In your center, in what percentage of patients do you perform a diaphragmatic peritonectomy? *	
a. 0%	56 (30%)
b. <25%	48 (26%)
c. 25-50%	36 (19%)
d. 51-75%	28 (15%)
e. 76-100%	18 (10%)
**34. In your center, in what percentage of patients do you perform a bowel resection?**	
a. < 5%	94 (49%)
b. 5-10%	56 (29%)
c. 10-20%	26 (14%)
d. >20%	16 (8%)
**35. In your center, in what percentage of patients do you perform a splenectomy?**	
a. <5%	126 (66%)
b. 5%-15%	50 (26%)
c. > 15%	16 (8%)
**36. In your center, in what percentage of patients do you perform a liver resection?**	
a. 0%	64 (33%)
b. 1-10%	118 (61%)
c. >10%	10 (5%)
**37. In your center, in what percentage of patients do you perform multiple liver resection?**	
a. 0%	126 (66%)
b. 1-10%	60 (31%)
c. >10%	6 (3%)
**38. In your center, in what percentage of patients do you perform a distal resection of the pancreas?**	
a. 0%	114 (59%)
b. 1-5%	64 (33%)
c. >5%	14 (7%)
**39. In your center, in what percentage of patients do you perform a cholecystectomy?**	
a. 0%	56 (29%)
b. 1-10%	118 (61%)
c. >10%	18 (9%)
**40. In your center, in what percentage of patients do you perform systematic pelvic lymphadenectomy?**	
a. 0%	52 (27%)
b. 1-25%	50 (26%)
c. 26-50%	42 (22%)
d. > 50%	48 (25%)
**41. In your center, in what percentage of patients do you perform a systematic lombo-aortic lymphadenectomy?**	
a. 0%	62 (32%)
b. 1-25%	68 (35%)
c. 26-50%	28 (15%)
d. >50%	34 (18%)
**42. In your center, in what percentage of patients do you perform only bulky lymph nodes removal?**	
a. <25%	44 (23%)
b. 25-50%	56 (29%)
c. 51-75%	34 (18%)
d. 76-100%	58 (30%)
**43. For patients not eligible for surgery, neoadjuvant chemotherapy is decided based on histological exam:**	
a. Yes, in the majority of cases	88 (46%)
b. Yes, always	96 (50%)
c. Often, citologic examination of ascitic fluid is sufficient	8 (4%)
**44. In your center, what percentage of patients do you refer to neoadjuvant chemotherapy?**	
a. <20%	54 (28%)
b. 20-30%	64 (33%)
c. 31-40%	38 (20%)
d. >40%	36 (19%)
**45. In your center, what type of neoadjuvant chemotherapy do your patients receive?**	
a. Carboplatin and placlitaxel	160 (83%)
b. Carboplatin alone	10 (5%)
c. Carboplatin and other drug	16 (8%)
d. Other drug combinations	6 (3%)
**46. In your center, how many cycles of neoadjuvant chemotherapy do your patients receive on average before surgery?**	
a. 3	122 (64%)
b. 4	14 (7%)
c. ≥5	56 (29%)
**48. The answers to these questions are based on?**	
a. Rough estimate	158 (82%)
b. Database	34 (18%)

**Table 2:** Influence of gynecological oncological practice on answers to survey questions

	Do you practice Gynecologic Oncology?							
	a. Occasionally (N=84)		b. It is my principal activity (N=108)			Total (N=192)		*P* value
**4. How old are you?**								0.352
a. < 30 years	0 (0%)		0 (0%)			0 (0%)		
b. 30-35 years	14 (17%)		10 (9%)			24 (12%)		
c. 36-40 years	14 (17%)		14 (13%)			28 (15%)		
d. 41-50 years	20 (24%)		30 (28%)			50 (26%)		
e. > 50 years	36 (43%)		54 (50%)			90 (47%)		
**5. How many years of practice do you have?**								0.003
a. ≤ 5 years	16 (19%)		12 (11%)			28 (15%)		
b. 6-10 years	14 (17%)		4 (4%)			18 (9%)		
c. 11-15 years	10 (12%)		16 (15%)			26 (14%)		
d. > 15 years	44 (52%)		76 (70%)			120 (62%)		
**7. Do you perform EOC surgery as:**								< 0.001
a. First surgeon	30 (36%)		80 (74%)			110 (57%)		
b. Assistant surgeon	54 (64%)		28 (26%)			82 (43%)		
**8. How long have you been practicing oncological gynecology?**								0.004
a. ≤ 5 years	28 (33%)		16 (15%)			44 (23%)		
b. 6-10 years	12 (14%)		24 (22%)			36 (19%)		
c. > 10 years	44 (52%)		68 (63%)			112 (58%)		
**9. In your center, how many ovarian cancer patients are treated each year?**								< 0.001
a. ≤10	44 (52%)		8 (7%)			52 (27%)		
b. 11-20	22 (26%)		26 (24%)			48 (25%)		
c. 21-30	8 (10%)		12 (11%)			20 (10%)		
d. >30	10 (12%)		62 (57%)			72 (38%)		
**10. In your center, how many ovarian cancer patients at stage I-II are treated each year?**								0.503
a. ≤5/year	32 (38%)			34 (31%)		66 (34%)		
b. 6-10/ year	32 (38%)			42 (39%)		74 (39%)		
c. 11-15/year	8 (10%)			18 (17%)		26 (14%)		
d. 15/ year	12 (14%)			14 (13%)		26 (14%)		
**11. In your center, which surgical approach do you use in patients with early ovarian cancer?**								0.601
a. Laparoscopic/both	64 (76%)		86 (80%)			150 (78%)		
b. Laparotomic	20 (24%)		22 (20%)			42 (22%)		
**12. In your center, in what percentage of cases do you use the laparoscopic approach?**								0.016
a. ≤10	32 (38%)		24 (22%)			56 (29%)		
b. 11-30	16 (19%)		38 (35%)			54 (28%)		
c. > 30%	36 (43%)		46 (43%)			82 (43%)		
**14. In your center, how many ovarian cancer patients are candidated to fertility sparing surgery each year?**								0.002
a. 0-2/year	62 (74%)		58 (54%)			120 (62%)		
c. 2-5/year	16 (19%)		46 (43%)			62 (32%)		
b. > 5/year	6 (7%)		4 (4%)			10 (5%)		
**18. In your center, which surgical approach do you use in patients with ovarian tumors who are candidates for fertility sparing surgery?**								0.362
a. Laparoscopic/both	72 (86%)		98 (91%)			170 (89%)		
b. Laparotomic	12 (14%)		10 (9%)			22 (11%)		
19. In your center, in what percentage of cases do you adopt the laparoscopic approach in patients candidated for fertility sparing surgery?*								0.007
a. 0%	18 (22%)		8 (8%)			26 (14%)		
b. < 25%	22 (27%)		38 (37%)			60 (33%)		
c. 25-50%	10 (12%)		12 (12%)			22 (12%)		
d. 50-100%	16 (20%)		34 (33%)			50 (27%)		
e. 100%	16 (20%)		10 (10%)			26 (14%)		
**21. In your center, do you have the opportunity to perform an extemporaneous intraoperative examination?**								0.006
a. Yes	78 (93%)		108 (100%)			186 (97%)		
b. No	6 (7%)		0 (0%)			6 (3%)		
**22. In your center, do you have a dedicated pathologist available?**								< 0.001
a. Yes	40 (48%)		86 (80%)			126 (66%)		
b. No	44 (52%)		22 (20%)			66 (34%)		
**23. Do you have a dedicated general surgeon on your team?**								0.002
a. Yes	44 (52%)		80 (74%)				124 (65%)	
b. No	40 (48%)		28 (26%)				68 (35%)	
**24. What do you consider as optimal cytoduction?**								< 0.001
a. No gross residual disease	56 (67%)		96 (89%)				152 (79%)	
b. Residual disease ≤ 0.5 cm	12 (14%)		8 (7%)				20 (10%)	
c. Residual disease ≤ 1 cm	16 (19%)		4 (4%)				20 (10%)	
**25. In your center, who evaluate the residual disease after the surgery?**								0.319
a. The first operator	60 (75%)			78 (75%)			138 (75%)	
b. A second surgeon	2 (2%)			0 (0%)			2 (1%)	
c. The patient undergoes a CT scan after the surgery	18 (22%)			26 (25%)			44 (24%)	
**26. In your center, in what percentage of patients do you get optimal cytoreduction?**								< 0.001
a. <20%	26 (31%)			8 (7%)			34 (18%)	
b. 21-40%	22 (26%)			12 (11%)			34 (18%)	
c. 41-60%	14 (17%)			36 (33%)			50 (26%)	
d. 61-80%	14 (17%)			26 (24%)			40 (21%)	
e. >80%	8 (10%)			26 (24%)			34 (18%)	
**28. In your center, in cases where you suspect the impossibility of direct cytoreduction, do you always perform a diagnostic laparoscopy before laparotomy?**								< 0.001
a. Yes	58 (69%)				96 (89%)		154 (80%)	
b. No	26 (31%)				12 (11%)		38 (20%)	
**29. In your center, in cases where you suspect the impossibility of direct cytoreduction, do you always perform a minilaparotomy before laparotomy?**								1.000
a. Yes	12 (14%)				16 (15%)	28 (15%)		
b. No	72 (86%)				92 (85%)	164 (85%)		
**31. In your center, in what percentage of patients do you perform a diaphragmatic resection?**								< 0.001
a. 0%	58 (69%)				30 (28%)	88 (46%)		
b. 1%-20%	20 (24%)				56 (52%)	76 (40%)		
c. 21-40%	0 (0%)				12 (11%)	12 (6%)		
d. >40%	6 (7%)				10 (9%)	16 (8%)		
32. In your center, in what percentage of patients do you perform a diaphragmatic peritonectomy?*								< 0.001
a. 0%	44 (55%)	12 (11%)				56 (30%)		
b. <25%	20 (25%)	28 (26%)				48 (26%)		
c. 25-50%	6 (8%)	30 (28%)				36 (19%)		
d. 51-75%	4 (5%)	24 (23%)				28 (15%)		
e. 76-100%	6 (8%)	12 (11%)				18 (10%)		
**34. In your center, in what percentage of patients do you perform a bowel resection?**								< 0.001
a. < 5%	64 (76%)	30 (28%)				94 (49%)		
b. 5-10%	18 (21%)	38 (35%)				56 (29%)		
c. 10-20%	2 (2%)	24 (22%)				26 (14%)		
d. >20%	0 (0%)	16 (15%)				16 (8%)		
**35. In your center, in what percentage of patients do you perform a splenectomy?**								< 0.001
a. <5%	70 (83%)	56 (52%)				126 (66%)		
b. 5%-15%	10 (12%)	40 (37%)				50 (26%)		
c. > 15%	4 (5%)	12 (11%)				16 (8%)		
**36. In your center, in what percentage of patients do you perform a liver resection?**								< 0.001
a. 0%	50 (60%)	14 (13%)				64 (33%)		
b. 1-10%	32 (38%)	86 (80%)				118 (61%)		
c. >10%	2 (2%)	8 (7%)				10 (5%)		
**37. In your center, in what percentage of patients do you perform multiple liver resection?**								0.011
a. 0%	64 (76%)	62 (57%)				126 (66%)		
b. 1-10%	20 (24%)	40 (37%)				60 (31%)		
c. >10%	0 (0%)	6 (6%)				6 (3%)		
**38. In your center, in what percentage of patients do you perform a distal resection of the pancreas?**								< 0.001
a. 0%	64 (76%)	50 (46%)				114 (59%)		
b. 1-5%	0 (0%)	14 (13%)				14 (7%)		
c. >5%	20 (24%)	44 (41%)				64 (33%)		
**39. In your center, in what percentage of patients do you perform a cholecystectomy?**								< 0.001
a. 0%	38 (45%)	18 (17%)				56 (29%)		
b. 1-10%	38 (45%)	80 (74%)				118 (61%)		
c. >10%	8 (10%)	10 (9%)				18 (9%)		
**40. In your center, in what percentage of patients do you perform systematic pelvic lymphadenectomy?**								< 0.001
a. 0%	12 (14%)	40 (37%)				52 (27%)		
b. 1-25%	20 (24%)	30 (28%)				50 (26%)		
c. 26-50%	20 (24%)	22 (20%)				42 (22%)		
d. > 50%	32 (38%)	16 (15%)				48 (25%)		
**41. In your center, in what percentage of patients do you perform a systematic lombo-aortic lymphadenectomy?**								< 0.001
a. 0%	14 (17%)	48 (44%)				62 (32%)		
b. 1-25%	38 (45%)	30 (28%)				68 (35%)		
c. 26-50%	14 (17%)	14 (13%)				28 (15%)		
d. >50%	18 (21%)	16 (15%)				34 (18%)		
**42. In your center, in what percentage of patients do you perform only bulky lymph nodes removal?**								< 0.001
a. <25%	32 (38%)	12 (11%)				44 (23%)		
b. 25-50%	32 (38%)	24 (22%)				56 (29%)		
c. 51-75%	8 (10%)	26 (24%)				34 (18%)		
d. 76-100%	12 (14%)	46 (43%)				58 (30%)		
**43. For patients not eligible for surgery, neoadjuvant chemotherapy is decided based on histological exam:**								0.003
a. Yes, in the majority of cases	38 (45%)	50 (46%)				88 (46%)		
b. Yes, always	38 (45%)	58 (54%)				96 (50%)		
c. Often, citologic examination of ascitic fluid is sufficient	8 (10%)	0 (0%)				8 (4%)		
**44. In your center, what percentage of patients do you refer to neoadjuvant chemotherapy?**								0.078
a. <20%	24 (29%)	30 (28%)				54 (28%)		
b. 20-30%	26 (31%)	38 (35%)				64 (33%)		
c. 31-40%	12 (14%)	26 (24%)				38 (20%)		
d. >40%	22 (26%)	14 (13%)				36 (19%)		
**45. In your center, what type of neoadjuvant chemotherapy do your patients receive?**								< 0.001
a. Carboplatin and placlitaxel	58 (69%)	102 (94%)				160 (83%)		
b. Carboplatin alone	8 (10%)	2 (2%)				10 (5%)		
c. Carboplatin and other drug	14 (17%)	2 (2%)				16 (8%)		
d. Other drug combinations	4 (5%)	2 (2%)				6 (3%)		
**46. In your center, how many cycles of neoadjuvant chemotherapy do your patients receive on average before surgery?**								< 0.001
a. 3	36 (43%)	86 (80%)				122 (64%)		
b. 4	8 (10%)	6 (6%)				14 (7%)		
c. ≥5	40 (48%)	16 (15%)				56 (29%)		
**48. The answers to these questions are based on?**								< 0.001
a. Rough estimate	82 (98%)	76 (70%)				158 (82%)		
b. Database	2 (2%)	32 (30%)				34 (18%)		
								

## Abbreviations

EOC: epithelial ovarian cancer
OS: overall survival
AGENAS: The Italian National Agency for Regional Healthcare Services
MITO: Multicenter Italian Trials in Ovarian Cancer and Gynecologic Malignancies
AOGOI: Italian Association of the Ostetricians and Gynecologists
GGs: General Gynecologists
GOs: gynecologists with specific interests in gynecologic oncology
AS: assistant
RD: residual disease
CHT: chemotherapy
FS: first surgeon
