# Supplementary material for: An Italian National Survey on Ovarian Cancer Treatment at first diagnosis. There's None so Deaf as those who will not Hear
Source: J Cancer. 2021 May 27;12(15):4443–54. doi: 10.7150/jca.57894 (PMC8210549; doi:10.7150/jca.57894)
Supplement: Supplementary file 1 — Supplementary tables. [file jcav12p4443s1.pdf]

Supplementary Tables

Table S1. Frequency distribution of survey questions and answers of all physicians.

	(N=226)
**1. Where do you work ?**	
a. General Hospital	130 (58%)
b. University Hospital	62 (27%)
c. Research Institute	34 (15%)
d. Private Clinic	0 (0%)
**2. In which area of Italy do you work?**	
a. Norther Italy	104 (46%)
b. Central Italy	32 (14%)
c. Southern Italy	90 (40%)
**3. You are:**	
a. Resident	34 (15%)
b. Specialist	192 (85%)
**4. How old are you?**	
a. < 30 years	14 (6%)
b. 30-35 years	44 (19%)
c. 36-40 years	28 (12%)
d. 41-50 years	50 (22%)
e. > 50 years	90 (40%)
**5. How many years of practice do you have?**	
a. ≤ 5 years	60 (27%)
b. 6-10 years	18 (8%)
c. 11-15 years	26 (12%)
d. > 15 years	122 (54%)
**6. Do you practice Gynecologic Oncology?**	
a. Occasionally	106 (47%)
b. It is my principal activity	120 (53%)
**7. Do you perform EOC surgery as:**	
a. First surgeon	112 (50%)
b. Assistant surgeon	114 (50%)
**8. How long have you been practicing oncological gynecology?**	
a. ≤ 5 years	70 (31%)
b. 6-10 years	42 (19%)
c. > 10 years	114 (50%)
**9. In your center, how many ovarian cancer patients are treated each year?**	
a. <=10	66 (29%)
b. 11-20	54 (24%)

c. 21-30	24 (11%)
d. >30	82 (36%)

10. In your center, how many ovarian cancer patients at stage I-II are treated each year?

a. ≤5/year	74 (33%)
b. 6-10/ year	86 (38%)
c. 11-15/year	36 (16%)
d. 15/ year	30 (13%)

11. In your center, which surgical approach do you use in patients with early ovarian cancer?

a. Laparoscopic/both	172 (76%)
b. Laparotomic	54 (24%)

12. In your center, in what percentage of cases do you use the laparoscopic approach?

a. <=10	70 (31%)
b. 11-30	70 (31%)
c. > 30%	86 (38%)

14. In your center, how many ovarian cancer patients are candidated to fertility sparing surgery each year?

a. 0-2/year	140 (62%)
c. 2-5/year	70 (31%)
b. > 5/year	16 (7%)

18. In your center, which surgical approach do you use in patients with ovarian tumors who are candidates for fertility sparing surgery?

a. Laparoscopic/both	198 (88%)
b. Laparotomic	28 (12%)

19. In your center, in what percentage of cases do you adopt the laparoscopic approach in patients candidated for fertility sparing surgery? *

a. 0%	32 (15%)
b. < 25%	72 (33%)
c. 25-50%	32 (15%)
d. 50-100%	52 (24%)
e. 100%	28 (13%)

21. In your center, do you have the opportunity to perform an extemporaneous intraoperative examination?

a. Yes	220 (97%)
b. No	6 (3%)

22. In your center, do you have a dedicated pathologist available?

a. Yes	154 (68%)
b. No	72 (32%)

23. Do you have a dedicated general surgeon on your team?

a. Yes	144 (64%)
b. No	82 (36%)

24. What do you consider as optimal cytoduction?

a. No gross residual disease	180 (80%)
b. Residual disease ≤ 0.5 cm	24 (11%)
c. Residual disease ≤ 1 cm	22 (10%)

25. In your center, who evaluate the residual disease after the surgery?

a. The first operator	162 (74%)
b. A second surgeon	2 (1%)
c. The patient undergoes a CT scan after the surgery	54 (25%)

26. In your center ,in what percentage of patients do you get optimal cytoreduction?

a. <20%	40 (18%)
b. 21-40%	36 (16%)
c. 41-60%	64 (28%)
d. 61-80%	46 (20%)
e. >80%	40 (18%)

28. In your center, in cases where you suspect the impossibility of direct cytoreduction, do you always perform a diagnostic laparoscopy before laparotomy?

a. Yes	182 (81%)
b. No	44 (19%)

29. In your center, in cases where you suspect the impossibility of direct cytoreduction, do you always perform a minilaparotomy before laparotomy?

a. Yes	30 (13%)
b. No	196 (87%)

31. In your center,in what percentage of patients do you perform a diaphragmatic resection?

a. 0%	102 (45%)
b. 1%-20%	92 (41%)
c. 21-40%	14 (6%)
d. >40%	18 (8%)

32. In your center, in what percentage of patients do you perform a diaphragmatic peritonectomy? *

a. 0%	66 (30%)
b. <25%	64 (29%)
c. 25-50%	38 (17%)
d. 51-75%	34 (15%)
e. 76-100%	18 (8%)

34. In your center, in what percentage of patients do you perform a bowel resection?

a. < 5%	114 (50%)
b. 5-10%	60 (27%)
c. 10-20%	30 (13%)
d. >20%	22 (10%)

35. In your center, in what percentage of patients do you perform a splenectomy?

a. <5%	150 (66%)
b. 5%-15%	58 (26%)
c. > 15%	18 (8%)

36. In your center, in what percentage of patients do you perform a liver resection?

a. 0%	76 (34%)
b. 1-10%	138 (61%)
c. >10%	12 (5%)

37. In your center, in what percentage of patients do you perform multiple liver resection?

a. 0%	144 (64%)
b. 1-10%	74 (33%)
c. >10%	8 (4%)

38. In your center, in what percentage of patients do you perform a distal resection of the pancreas?

a. 0%	136 (60%)
b. 1-5%	74 (33%)
c. >5%	16 (7%)

39. In your center, in what percentage of patients do you perform a cholecystectomy?

a. 0%	66 (29%)
b. 1-10%	138 (61%)
c. >10%	22 (10%)

40. In your center, in what percentage of patients do you perform systematic pelvic lymphadenectomy?

a. 0%	58 (26%)
b. 1-25%	56 (25%)
c. 26-50%	46 (20%)
d. > 50%	66 (29%)

41. In your center, in what percentage of patients do you perform a systematic lombo-aortic lymphadenectomy?

a. 0%	70 (31%)
b. 1-25%	74 (33%)

c. 26-50% — 36 (16%)
d. >50% — 46 (20%)

42. In your center, In what percentage of patients do you perform only bulky lymph nodes removal?

a. <25%	64 (28%)
b. 25-50%	62 (27%)
c. 51-75%	36 (16%)
d. 76-100%	64 (28%)

43. For patients not eligible for surgery, neoadjuvant chemotherapy is decided based on histological exam:

a. Yes, in the majority of cases	114 (50%)
b. Yes, always	104 (46%)
c. Often, citologic examination of ascitic fluid is sufficient	8 (4%)

44. In your center, what percentage of patients do you refer to neoadjuvant chemotherapy?

a. <20%	58 (26%)
b. 20-30%	74 (33%)
c. 31-40%	52 (23%)
d. >40%	42 (19%)

45. In your center, what type of neoadjuvant chemotherapy do your patients receive?

a. Carboplatin and placlitaxel	194 (86%)
b. Carboplatin alone	10 (4%)
c. Carboplatin and other drug	16 (7%)
d. Other drug combinations	6 (3%)

46. . In your center, how many cycles of neoadjuvant chemotherapy do your patients receive on average before surgery?

a. 3	122 (64%)
b. 4	14 (7%)
c. >=5	56 (29%)

48. The answers to these questions are based on?

a. Rough estimate	190 (84%)
b. Database	36 (16%)

Table S2. Frequency distribution of survey questions and answers among first operators and assistants.

	7. Do you perform EOC surgery as: a. First surgeon (N=110)	7. Do you perform EOC surgery as: b. Assistant surgeon (N=82)	Total (N=192)	p value
**4. How old are you?**				< 0.001
a. < 30 years	2 (2%)	22 (27%)	24 (12%)	
b. 30-35 years	10 (9%)	18 (22%)	28 (15%)	
c. 36-40 years	26 (24%)	24 (29%)	50 (26%)	
d. 41-50 years	72 (65%)	18 (22%)	90 (47%)	
e. > 50 years				< 0.001
**5. How many years of practice do you have?**	2 (2%)	26 (32%)	28 (15%)	
a. ≤ 5 years	2 (2%)	16 (20%)	18 (9%)	
b. 6-10 years	12 (11%)	14 (17%)	26 (14%)	
c. 11-15 years	94 (85%)	26 (32%)	120 (62%)	
**6. Do you practice Gynecologic Oncology?**				< 0.001
a. Occasionally	30 (27%)	54 (66%)	84 (44%)	
b. It is my principal activity	80 (73%)	28 (34%)	108 (56%)	
**8. How long have you been practicing oncological gynecology?**				< 0.001
a. ≤ 5 years	12 (11%)	32 (39%)	44 (23%)	
b. 6-10 years	14 (13%)	22 (27%)	36 (19%)	
c. > 10 years	84 (76%)	28 (34%)	112 (58%)	
**9. In your center, how many ovarian cancer patients are treated each year?**				0.045
a. <=10	22 (20%)	30 (37%)	52 (27%)	
b. 11-20	32 (29%)	16 (20%)	48 (25%)	
c. 21-30	10 (9%)	10 (12%)	20 (10%)	
d. >30	46 (42%)	26 (32%)	72 (38%)	
**10. In your center, how many ovarian cancer patients at stage I-II are treated each year?**				0.181
a. ≤5/year	44 (40%)	22 (27%)	66 (34%)	
b. 6-10/ year	36 (33%)	38 (46%)	74 (39%)	
c. 11-15/year	16 (15%)	10 (12%)	26 (14%)	
d. 15/ year	14 (13%)	12 (15%)	26 (14%)	
**11. In your center, which surgical**				0.162

**approach do you use in patients with early ovarian cancer?**				
a. Laparoscopic/both	90 (82%)	60 (73%)	150 (78%)	
b. Laparotomic	20 (18%)	22 (27%)	42 (22%)	
**12. In your center, in what percentage of cases do you use the laparoscopic approach?**				0.043
a. <=10	24 (22%)	32 (39%)	56 (29%)	
b. 11-30	34 (31%)	20 (24%)	54 (28%)	
c. > 30%	52 (47%)	30 (37%)	82 (43%)	
**14. In your center, how many ovarian cancer patients are candidated to fertility sparing surgery each year?**				0.002
a. 0-2/year	60 (55%)	60 (73%)	120 (62%)	
c. 2-5/year	46 (42%)	16 (20%)	62 (32%)	
b. > 5/year	4 (4%)	6 (7%)	10 (5%)	
**18. In your center, which surgical approach do you use in patients with ovarian tumors who are candidates for fertility sparing surgery?**				0.258
a. Laparoscopic/both	100 (91%)	70 (85%)	170 (89%)	
b. Laparotomic	10 (9%)	12 (15%)	22 (11%)	
**19. In your center, in what percentage of cases do you adopt the laparoscopic approach in patients candidated for fertility sparing surgery? ***				0.010
a. 0%	8 (7%)	18 (24%)	26 (14%)	
b. < 25%	42 (39%)	18 (24%)	60 (33%)	
c. 25-50%	10 (9%)	12 (16%)	22 (12%)	
d. 50-100%	30 (28%)	20 (26%)	50 (27%)	
e. 100%	18 (17%)	8 (11%)	26 (14%)	
**21. In your center, do you have the opportunity to perform an extemporaneous intraoperative examination?**				1.000
a. Yes	106 (96%)	80 (98%)	186 (97%)	
b. No	4 (4%)	2 (2%)	6 (3%)	
**22. In your center, do you have a dedicated pathologist available?**				0.283
a. Yes	76 (69%)	50 (61%)	126 (66%)	
b. No	34 (31%)	32 (39%)	66 (34%)	
**23. Do you have a dedicated general surgeon on your team?**				0.001
a. Yes	82 (75%)	42 (51%)	124 (65%)	

b. No	28 (25%)	40 (49%)	68 (35%)	
**24. What do you consider as optimal cytoduction?**				0.159
a. No gross residual disease	88 (80%)	64 (78%)	152 (79%)	
b. Residual disease ≤ 0.5 cm	14 (13%)	6 (7%)	20 (10%)	
c. Residual disease ≤ 1 cm	8 (7%)	12 (15%)	20 (10%)	
**25. In your center, who evaluate the residual disease after the surgery?**				0.246
a. The first operator	84 (78%)	54 (71%)	138 (75%)	
b. A second surgeon	2 (2%)	0 (0%)	2 (1%)	
c. The patient undergoes a CT scan after the surgery	22 (20%)	22 (29%)	44 (24%)	
**26. In your center, in what percentage of patients do you get optimal cytoreduction?**				0.002
a. <20%	16 (15%)	18 (22%)	34 (18%)	
b. 21-40%	14 (13%)	20 (24%)	34 (18%)	
c. 41-60%	36 (33%)	14 (17%)	50 (26%)	
d. 61-80%	18 (16%)	22 (27%)	40 (21%)	
e. >80%	26 (24%)	8 (10%)	34 (18%)	
**28. In your center, in cases where you suspect the impossibility of direct cytoreduction, do you always perform a diagnostic laparoscopy before laparotomy?**				0.044
a. Yes	94 (85%)	60 (73%)	154 (80%)	
b. No	16 (15%)	22 (27%)	38 (20%)	
**29. In your center, in cases where you suspect the impossibility of direct cytoreduction, do you always perform a minilaparotomy before laparotomy?**				1.000
a. Yes	16 (15%)	12 (15%)	28 (15%)	
b. No	94 (85%)	70 (85%)	164 (85%)	
**31. In your center,in what percentage of patients do you perform a diaphragmatic resection?**				< 0.001
a. 0%	38 (35%)	50 (61%)	88 (46%)	
b. 1%-20%	48 (44%)	28 (34%)	76 (40%)	
c. 21-40%	12 (11%)	0 (0%)	12 (6%)	
d. >40%	12 (11%)	4 (5%)	16 (8%)	
**32. In your center, in what percentage of patients do you perform a diaphragmatic peritonectomy? ***				< 0.001
a. 0%	24 (22%)	32 (42%)	56 (30%)	

b. <25%	22 (20%)	26 (34%)	48 (26%)	
c. 25-50%	26 (24%)	10 (13%)	36 (19%)	
d. 51-75%	22 (20%)	6 (8%)	28 (15%)	
e. 76-100%	16 (15%)	2 (3%)	18 (10%)	
**34. In your center, in what percentage of patients do you perform a bowel resection?**				< 0.001
a. < 5%	36 (33%)	58 (71%)	94 (49%)	
b. 5-10%	42 (38%)	14 (17%)	56 (29%)	
c. 10-20%	18 (16%)	8 (10%)	26 (14%)	
d. >20%	14 (13%)	2 (2%)	16 (8%)	
**35. In your center, in what percentage of patients do you perform a splenectomy?**				< 0.001
a. <5%	60 (55%)	66 (80%)	126 (66%)	
b. 5%-15%	38 (35%)	12 (15%)	50 (26%)	
c. > 15%	12 (11%)	4 (5%)	16 (8%)	
**36. In your center, in what percentage of patients do you perform a liver resection?**				< 0.001
a. 0%	22 (20%)	42 (51%)	64 (33%)	
b. 1-10%	82 (75%)	36 (44%)	118 (61%)	
c. >10%	6 (5%)	4 (5%)	10 (5%)	
**37. In your center, in what percentage of patients do you perform multiple liver resection?**				0.360
a. 0%	76 (69%)	50 (61%)	126 (66%)	
b. 1-10%	30 (27%)	30 (37%)	60 (31%)	
c. >10%	4 (4%)	2 (2%)	6 (3%)	
**38. In your center, in what percentage of patients do you perform a distal resection of the pancreas?**				0.005
a. 0%	56 (51%)	58 (71%)	114 (59%)	
b. 1-5%	42 (38%)	22 (27%)	64 (33%)	
c. >5%	12 (11%)	2 (2%)	14 (7%)	
**39. In your center, in what percentage of patients do you perform a cholecystectomy?**				0.038
a. 0%	26 (24%)	30 (37%)	56 (29%)	
b. 1-10%	76 (69%)	42 (51%)	118 (61%)	
c. >10%	8 (7%)	10 (12%)	18 (9%)	
**40. In your center, in what percentage of patients do you perform systematic pelvic lymphadenectomy?**				0.018

a. 0%	34 (31%)	18 (22%)	52 (27%)	
b. 1-25%	30 (27%)	20 (24%)	50 (26%)	
c. 26-50%	28 (25%)	14 (17%)	42 (22%)	
d. > 50%	18 (16%)	30 (37%)	48 (25%)	
**41. In your center, in what percentage of patients do you perform a systematic lombo-aortic lymphadenectomy?**				0.016
a. 0%	40 (36%)	22 (27%)	62 (32%)	
b. 1-25%	44 (40%)	24 (29%)	68 (35%)	
c. 26-50%	14 (13%)	14 (17%)	28 (15%)	
d. >50%	12 (11%)	22 (27%)	34 (18%)	
**42. In your center, In what percentage of patients do you perform only bulky lymph nodes removal?**				< 0.001
a. <25%	14 (13%)	30 (37%)	44 (23%)	
b. 25-50%	32 (29%)	24 (29%)	56 (29%)	
c. 51-75%	22 (20%)	12 (15%)	34 (18%)	
d. 76-100%	42 (38%)	16 (20%)	58 (30%)	
**43. For patients not eligible for surgery, neoadjuvant chemotherapy is decided based on histological exam:**				0.002
a. Yes, in the majority of cases	56 (51%)	32 (39%)	88 (46%)	
b. Yes, always	54 (49%)	42 (51%)	96 (50%)	
c. Often, citologic examination of ascitic fluid is sufficient	0 (0%)	8 (10%)	8 (4%)	
**44. In your center, what percentage of patients do you refer to neoadjuvant chemotherapy?**				0.011
a. <20%	22 (20%)	32 (39%)	54 (28%)	
b. 20-30%	42 (38%)	22 (27%)	64 (33%)	
c. 31-40%	20 (18%)	18 (22%)	38 (20%)	
d. >40%	26 (24%)	10 (12%)	36 (19%)	
**45. In your center, what type of neoadjuvant chemotherapy do your patients receive?**				0.006
a. Carboplatin and placlitaxel	100 (91%)	60 (73%)	160 (83%)	
b. Carboplatin alone	4 (4%)	6 (7%)	10 (5%)	
c. Carboplatin and other drug	4 (4%)	12 (15%)	16 (8%)	
d. Other drug combinations	2 (2%)	4 (5%)	6 (3%)	
**46. In your center, how many cycles of neoadjuvant chemotherapy do your patients receive on average before surgery?**				0.027

a. 3	78 (71%)	44 (54%)	122 (64%)	
b. 4	8 (7%)	6 (7%)	14 (7%)	
c. >=5	24 (22%)	32 (39%)	56 (29%)	
**48. The answers to these questions are based on?**				0.090
a. Rough estimate	86 (78%)	72 (88%)	158 (82%)	
b. Database	24 (22%)	10 (12%)	34 (18%)	
